# Exploring unusual bodily experiences, basic self disturbances and multimodal hallucinations in the non-clinical population: a cross-sectional study

**DOI:** 10.1192/bjo.2021.784

**Published:** 2021-06-18

**Authors:** Lucretia Thomas, Renate Reniers, Lénie Torregrossa, Clara Humpston

**Affiliations:** 1 Medical School, College of Medical and Dental Sciences, University of Birmingham; 2 Institute of Clinical Sciences, University of Birmingham, Institute for Mental Health, School of Psychology, University of Birmingham, Centre for Human Brain Health, University of Birmingham; 3 Department of Psychology, Vanderbilt University; 4 Institute for Mental Health, School of Psychology, University of Birmingham

## Abstract

**Aims:**

Psychosis research has largely focused on symptoms which are easier to define. Symptoms which are challenging to detect and articulate, including disturbances in the basic- and bodily-self, may not be volunteered by patients, despite causing significant distress. Increased understanding of such symptoms, which may present in the prodromal phase of psychosis and persist following the remission of positive symptoms, may allow patients who experience these to be better supported.

This study aims to explore how disturbances in the basic- and bodily-self relate to multimodal hallucinations. Through sampling a non-clinical population, this study takes the continuum approach to psychosis, where individuals experience sub-clinical psychotic symptoms which do not cause distress or functional impairment.

It is hypothesised that individuals with greater hallucination proneness will exhibit greater severity of ambiguous and imprecise mapping of bodily experiences, and will report greater levels of basic and bodily-self disturbance. This project also aims to evaluate Audiograph as a newly developed tool for creating representations of visual hallucinations.

**Method:**

This is a two-stage cross-sectional study. In stage one, participants completed the Multi-Modality Unusual Sensory Experiences Questionnaire to assess hallucination-proneness. In stage two, all participants were invited to complete seven further validated questionnaires which assessed basic- and bodily self-disturbances alongside co-variates including anxiety and depression symptoms, delusion-proneness and loneliness. Participants also completed emBODY, a computer-based task which allows participants to map the bodily sensations they experience during 13 different emotional states. Participants with high-hallucination proneness also completed the Audiograph task. Hierarchical linear regression, conducted using Stata, will be used to model the influence of hallucination proneness on measures of basic- and bodily-self disturbance. MATLAB will be used to generate topographical maps of the data from emBODY; maps will be compared between different emotional states using linear discriminant analysis, and between high and low hallucination proneness groups using Spearman's test.

**Result:**

Currently, 50 of the 104 stage one participants have completed stage two.

Since this project comprises a compulsory component of the presenting author's intercalated degree, data collection will cease on the 29th of March in advance of their poster and write-up submission deadlines in May.

**Conclusion:**

Although basic- and bodily-self disturbances have been assessed in previous studies using various techniques, no single study has assessed these alongside multimodal hallucinations to link these concepts together as a whole, especially not in a general population sample. The added value of this project is to precisely address this gap in knowledge.

